# Randomized Video-Feedback Intervention in Home-Based Childcare: Improvement of Children’s Wellbeing Dependent on Time Spent with Trusted Caregiver

**DOI:** 10.1007/s10566-015-9344-8

**Published:** 2016-01-19

**Authors:** Marleen G. Groeneveld, Harriet J. Vermeer, Marinus H. van IJzendoorn, Mariëlle Linting

**Affiliations:** Centre for Child and Family Studies, Leiden University, P.O. Box 9555, 2300 RB Leiden, The Netherlands

**Keywords:** Children’s wellbeing, Home-based childcare, Randomized controlled trial, Video-based intervention, VIPP

## Abstract

**Background:**

The childcare environment offers a wide array of developmental opportunities for children. Providing children with a feeling of security to explore this environment is one of the most fundamental goals of childcare.

**Objective:**

In the current study the effectiveness of Video-feedback Intervention to promote Positive Parenting-Child Care (VIPP-CC) was tested on children’s wellbeing in home-based childcare in a randomized controlled trial.

**Methods:**

Forty-seven children and their caregivers were randomly assigned to the intervention group or control group. Children’s wellbeing, caregiver sensitivity, and global childcare quality were observed during a pretest and a posttest.

**Results:**

We did not find an overall intervention effect on child wellbeing, but a significant interaction effect with months spent with a trusted caregiver was present. Children who were less familiar with the caregiver showed an increase in wellbeing scores in both the intervention and control group, but for the group of children who were more familiar with the caregiver, wellbeing increased only in the intervention group.

**Conclusions:**

Although there was no overall effect of the VIPP-CC on children’s wellbeing, the VIPP-CC seems effective in children who have been cared for by the same trusted caregiver for a longer period of time.

## Introduction

A feeling of wellbeing is a necessary condition for children to effectively explore their environment and thus encounter developmental opportunities. The childcare environment offers a wide array of such developmental opportunities. Providing children with a feeling of security to explore this environment is one of the most fundamental goals of childcare. The current study matches this goal by focusing on a video-feedback intervention aimed at improving childcare quality and ultimately child wellbeing in home-based childcare.

A large number of children are attending childcare nowadays, and home-based childcare has become a commonly used type of care. The NICHD Early Child Care Research Network reported that 24 % of the children in their sample attended home-based childcare at entry in childcare (NICHD ECCRN 1998). In the Netherlands, 16 % of the children attending formal childcare visit home-based childcare (Statistics Netherlands 2014), in which a caregiver takes care of a small group of children in her own house. Children in home-based childcare are taken care of the entire day by one caregiver in the same environment. Therefore we expect that the improvement of childcare quality of this specific setting will positively influence the children’s wellbeing during the day at childcare.

### Children’s Wellbeing in Childcare

The concept of child wellbeing is crucial in childcare (Laevers 2000) and can be defined as the extent to which children feel safe, self-confident, relaxed, and are enjoying the activities they are involved in (Riksen-Walraven 2004). Children’s emotional wellbeing is one of the most important indicators of the quality of a childcare setting. Because it affects children’s emotional and physical development (Davis et al. 2010; Howard and McInnes 2012; Laevers 2000).

Previous studies showed that children’s wellbeing in childcare is related to different aspects of the quality of care. De Schipper et al. (2004) found that daily stability in childcare centers was related to children’s wellbeing reported by their caregivers: children who experienced more stable program features of the childcare environment and were enrolled in fewer care arrangements felt more at ease in the center, as reported by their caregivers. Also, children were rated higher on wellbeing when trusted caregivers were more available for contact. In addition, in a recent Spanish study, an association was reported between children’s observed wellbeing and caregiver–child ratio: children in larger groups scored lower on wellbeing (Barandiaran et al. 2015).

Caregiver sensitivity is another important aspect of childcare quality. Sensitivity is the ability to accurately perceive the child’s signals and to respond promptly and adequately to these signals (Ainsworth et al. 1978). By minding these signals, caregivers may incorporate stimulation of social-emotional development as well as cognitive development in their daily routines, without overstimulating children. In home-based childcare, caregiver sensitivity has been associated with children’s observed wellbeing: children with more sensitive caregivers showed a higher wellbeing, and displayed lower cortisol levels (stress hormone) across the day than children with less sensitive caregivers (Groeneveld et al. 2010).

### Improving Caregiver–Child Interactions in Childcare

Thus, quality of care, and specifically quality of caregiver–child interactions, seems essential for children’s wellbeing. The question arises whether interventions aimed at improving caregiver–child interactions will affect child wellbeing as well. Although, in our view, wellbeing is one of the most fundamental aspects of all types of childcare, most interventions in childcare focus on other developmental outcomes in children. Interventions focusing on the improvement of caregiver behavior have shown to be successful in increasing children’s language development (Downer et al. 2011; Wasik et al. 2006), peer interaction (Girolametto et al. 2004; Snyder et al. 2011), and in decreasing children’s behavior problems (Barnett et al. 2008; Girard et al. 2011; NICHD ECCRN NICHD Early Child Care Research Network 2002; Rusby et al. 2008; Snyder et al. 2011). Interventions focusing on children’s wellbeing are underrepresented in the childcare literature. Werner et al. (2015) showed in their meta-analysis that targeted interventions focused on caregiver–child interactions are moderately effective in improving childcare quality on three levels: classroom quality (*k* = 11; Hedges’ *g* = 0.39), caregiver interaction skills (*k* = 10; Hedges’ *g* = 0.44), and, to a lesser extent, child behavior (*k* = 6; Hedges’ *g* = 0.26). Thus, the effect on the child level regarding social-emotional behavior was small, yet significant. The authors conclude that there is a lack of intervention studies using solid designs with sufficient power focusing on social-emotional outcomes in children. Although these studies did not measure ‘wellbeing’ per se, wellbeing is frequently used as an overarching term all of these domains (Amato and Keith 1991; Bradley and Vandell 2007; Clarke-Stewart and Hayward 1996).

Studies in home-based childcare, especially studies focusing on interventions to promote the social-emotional environment in childcare, are scarce. Three studies found positive effects of broad interventions on global quality, sensitivity or caregiver attitudes (Aguirre and Marshall 1988; Espinosa et al. 1999; Kontos et al. 1996), but in these studies no control group (Espinosa et al. 1999; Aguirre and Marshall 1988) or randomized control group (Kontos et al. 1996) was present. In a more recent study the effectiveness of an intervention (Carescapes program) in home-based childcare was tested in a randomized controlled trial (Rusby et al. 2008). The use of effective behavior management practices increased in the intervention group while behavior problems of the children decreased, but both effects did not maintain over time. For a broader overview of interventions in home-based care, see Groeneveld et al. (2012).

### Interventions to improve parent–child interactions

Programs aimed at enhancing the quality of mother–child interactions have been studied more often than programs directed at professional caregivers’ sensitivity. Bakermans-Kranenburg et al. (2003) conducted a meta-analysis of 80 studies to test the effectiveness of various types of interventions for enhancing maternal sensitivity. They showed that randomized interventions appeared effective in changing insensitive parenting (*d* = 0.33) and infant attachment insecurity (*d* = 0.20). Interventions with video-feedback were more effective (*d* = 0.44) than interventions without this method (*d* = 0.31). Interventions with fewer than five sessions were as effective (*d* = 0.42) as interventions with 5–16 sessions (*d* = 0.38), but interventions with more than 16 sessions were less effective (*d* = 0.21) than interventions with a smaller number of sessions. Based on this meta-analysis, a short-term, behaviorally focused intervention program was developed: the Video-feedback Intervention to promote Positive Parenting and Sensitive Discipline (VIPP-SD; Juffer et al. 2008). Based on both attachment theory (Ainsworth et al. 1978; Bowlby 1969) and coercion theory (Patterson 1982), the goal of VIPP-SD is to enhance parental sensitivity as well as sensitive discipline. Mother and child are videotaped during daily situations at home. Videotaped episodes are discussed with the mother, focusing on various parts of sensitivity as defined by Ainsworth et al. (1978). First, during the videotaped episodes the intervener focuses on observing the child’s signals in an accurate way. Second, through positive reinforcement of the mother’s sensitive behavior shown on the videotape, the mother is reinforced to respond to the child’s signals in an adequate and prompt way.

Studies using the VIPP-SD approach showed positive effects on maternal sensitivity in intervention groups compared to control groups in various samples: insecure mothers (Klein Velderman et al. 2006a, b), insensitive mothers (Kalinauskiene et al. 2009), mothers with eating disorders (Stein et al. 2006), adoptive mothers (Juffer et al. 2005), and mothers of children with externalizing problems (Van Zeijl et al. 2006; for an overview see Juffer et al. 2009). The effectiveness of the VIPP-SD in families has also been shown in changing children’s behavior. The intervention successfully decreased externalizing behavior problems in preschoolers in a high risk sample with an overrepresentation of insecure adult attachment representations (Klein Velderman et al. 2006a, b). The VIPP-SD proved to be especially effective in decreasing externalizing behavior in children with the Dopamine D4 receptor polymorphism, a polymorphism that is associated with motivational and reward mechanisms and Attention Deficit Hyperactivity Disorder in children (Bakermans-Kranenburg et al. 2003). It has also been shown that in families with more marital discord or more daily hassles, the VIPP-SD resulted in a decrease of overactive problem behaviors in the children (Van Zeijl et al. 2006). Until now, no studies have focused on the effectiveness of the VIPP-SD in improving children’s wellbeing.

### VIPP Child Care

The adaptation of the VIPP-SD for home-based childcare—the VIPP-ChildCare (VIPP-CC)—was described by Groeneveld et al. (2012). As the situation in home-based childcare differs from the home situation, the VIPP-SD was adapted to caregivers taking care of a group of children by slightly modifying the procedure and materials of the home visits. A randomized controlled trial in home-based childcare (Groeneveld et al. 2012) showed that the VIPP-CC improved global childcare quality (*d* = 0.76). In addition, although the expected increase in observed sensitivity was not found (*d* = 0.21), caregivers in the intervention group showed a more positive attitude towards sensitive caregiving and limit setting after the intervention than caregivers in the control group (*d* = 0.69).The current study uses the same dataset, but also includes child variables in the analyses. As suggested in the meta-analysis by Werner et al. (2015), there remains an urgent need for more randomized controlled trials with a solid design and high quality measures in order to shed more light on which childcare components for which children are most critical in supporting children’s socio-emotional development. The reported study can make an important contribution to the knowledge of the effects of interventions to promote children’s wellbeing in home-based childcare, using data from the previously reported study, which is a randomized controlled trial including observations of caregiver behavior as well as children’s behavior during childcare.

Familiarity with a caregiver may be an important moderator of this association. In the reported study, we will focus on the amount of months a child has spent with this specific trusted caregiver. It should be noted that the number of weekly hours in childcare, which in the Netherlands is usually between 16 and 24 h, is not taken into account. Thus far, previous studies did not report on the impact of the months spent with a trusted caregiver on intervention effects. If children attend childcare for a longer period of time, and are in the care of the same caregiver for a longer period of time, they may feel more at ease, and thus be more receptive to interventions. In the current study, we will focus on the effect of a video-feedback intervention on the wellbeing of children, taking months spent with the trusted caregiver into account.

### Aims of this Study

In the current paper, we will focus on the effect of the VIPP-CC on the wellbeing of the children in the childcare setting. We will also examine whether the intervention effect is moderated by childcare quality and months children spent with their trusted caregiver. We expect the intervention program to be effective in enhancing children’s wellbeing during childcare, especially for children in higher quality settings. In addition, if children, have been in the care of the same caregiver for a longer period of time, they may feel more at ease in childcare and thus be more receptive to the intervention. Following this line of reasoning, we expect children in the intervention group to show a larger increase in wellbeing when they have been in the care of their caregiver for a longer period of time.

## Methods

### Participants and Randomization

**Fig. 1:**
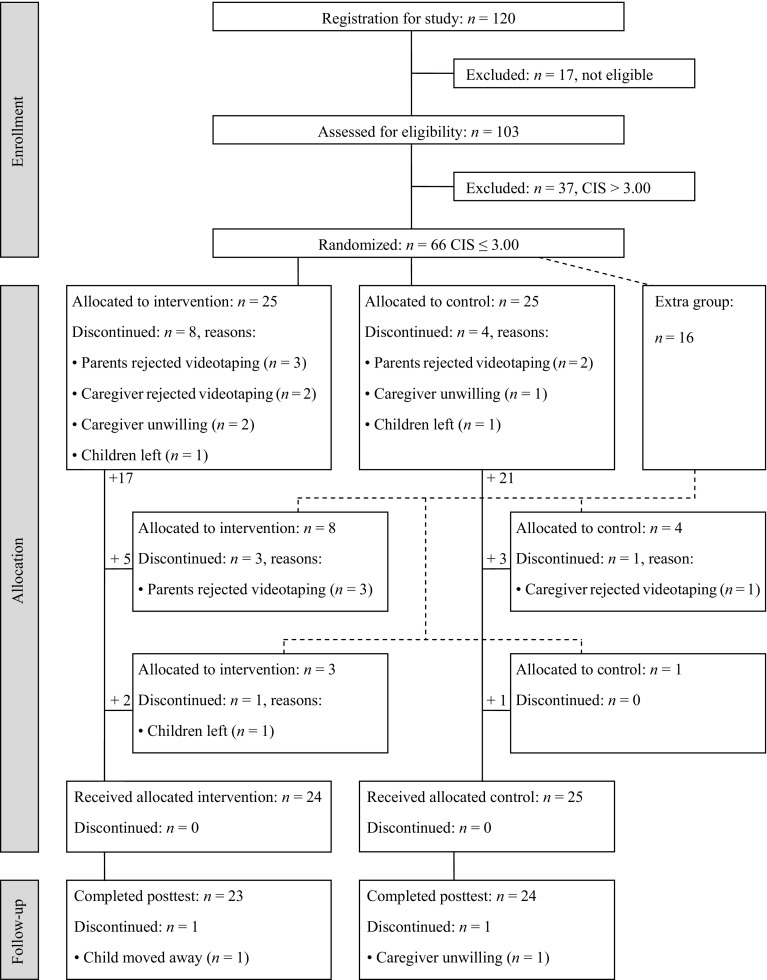
Flow Chart: caregiver–child pairs included in the RCT

Caregivers and children in this randomized, controlled study were recruited from 23 home-based childcare organizations in the western region of the Netherlands from both urban and rural areas. Inclusion criteria were: (1) caregivers took care of at least two children under the age of four, (2) caregivers were not biologically related to these children, and (3) caregiving took place in the caregiver’s own home. Invitation letters were sent to approximately 1000 caregivers. In total 157 caregivers refused to participate: at least 20 % refused because they did not meet the inclusion criteria described in the invitation letter. Other frequently mentioned reasons were that caregivers felt uncomfortable with video recordings or were too busy. Registration for the study was closed after agreement to participate was obtained from 120 caregivers. The flow chart (Fig. 1) shows participant progress through the phases of the randomized trial, which lasted for 6 months including selection (baseline), pretest assessment, intervention (or control condition), and posttest assessment. All measurements and the intervention took place at caregivers’ homes during childcare.

In September 2008, all caregivers were invited for the baseline visit. Seventeen caregivers were not eligible for the study, because inclusion criteria were not met. All other 103 caregivers were visited between November 2008 and January 2009 by an independent observer who measured caregiver sensitivity and global childcare quality for one of the children, the target child. This target child was randomly selected from the group, provided that he or she was no older than 38 months of age at the pretest, since the study period would take maximal 9 months and children leave childcare at the age of 48 months.

In the next step, we excluded caregivers who scored high on caregiver sensitivity (CIS > 3, *N* = 37). As a result, 66 caregivers were selected for our study. Based on our pilot study we anticipated a refusal rate of about 25 % (e.g., because of changes in the childcare arrangement, such as children leaving). Therefore, we included an extra group of caregivers to avoid selective attrition. The 66 caregivers were randomly assigned to either the intervention group (*n* = 25), the control group (*n* = 25), or the extra group (*n* = 16). Because of availability of interveners, the number of participants in the intervention group and the control group was restricted to 25. Following randomization procedures (random numbers), participants were assigned to one of these three groups. The 50 caregivers in the intervention group and the control group received a letter revealing whether they were assigned to the ‘training’ (intervention) or the ‘telephone’ (control) group. Eight caregivers in the intervention group and four caregivers in the control group refused to participate after receiving this letter. Caregivers from the extra group were randomly assigned to the intervention (*n* = 8) or the control group (*n* = 4). Of these caregivers, again four caregivers declined from the study, and caregivers from the extra group were again randomly assigned to the intervention (*n* = 3) or the control group (*n* = 1). Of this group, only one caregiver (in the intervention group) discontinued, because all the children she was taking care of had left. The caregivers who declined from the study (*n* = 17) did not differ on caregiver sensitivity from caregivers who remained in the study [*t* (63) = −1.66, *p* = .11]. In addition, caregivers who declined from the intervention group (*n* = 12) did not differ on caregiver sensitivity from caregivers who declined from the control group (*n* = 5) [*t* (15) = −0.34, *p* = .74]. The allocation phase resulted in two groups of caregivers: 24 caregivers in the intervention group and 25 caregivers in the control group.

All 49 caregivers received a pretest home visit, followed by the intervention or control period. The posttest took place in May–July 2009, after which the trial was ended. One caregiver in the control group did not complete the posttest because she cancelled all appointments. In addition, one of the target children in the intervention group left childcare, because his parents moved to another region of the Netherlands. Scores on all measures (global quality, sensitivity, and wellbeing) during baseline and pretest, did not differ from the other caregivers’ and children’s mean scores. The final sample included 47 caregiver–child pairs: 23 pairs in the intervention group and 24 pairs in the control group. Demographic information on the children is summarized in Table 1. Children’s age, months spent with caregiver and parental educational background did not differ significantly between the two groups. Mothers of children in the intervention group had on average 13.68 years (*SD* = 2.02) of education after primary school entrance, and mothers of children in control group had on average 13.50 years of education after age 6 [*SD* = 2.35, *t* (39) = −0.09, *p* = .93]. For fathers, no differences in level of education between the two types of care were present either [intervention group *M* = 12.21, *SD* = 2.62; control group *M* = 13.39, *SD* = 2.25, *t* (38) = −0.08, *p* = .94]. The mean age of the mothers did not differ between the two groups [intervention group: *M* = 32.90, *SD* = 5.25, control group: *M* = 33.05, *SD* = 5.35, *t* (35) = −0.26, *p* = .80], neither did the mean age of fathers [intervention group: *M* = 35.81, *SD* = 5.00, control group: *M* = 35.95, *SD* = 5.84, *t* (35) = 1.47, *p* = .15]. The family structure across the two groups of children was also similar: All children were raised in two-parent families. The nationality of almost all parents was Dutch (intervention group: mothers 94.7 % and fathers 94.7 %; control group: mothers 88.9 % and fathers 94.7 %). As described in Groeneveld et al. 2012), caregivers’ demographic backgrounds (e.g., age, education, number of working hours per week, group size) did not differ significantly between the two groups either. In addition, children’s and caregivers’ demographic backgrounds were comparable to a sample of a previous Dutch study in childcare homes (Groeneveld et al. 2012).

**Table 1:** Descriptive statistics for intervention group and control group during baseline/pretest and posttest

	Intervention group			Control group			Difference	
	*M*	*SD*	*SE*	*M*	*SD*	*SE*	*t*	*p*
Age children (months)	26.75	9.56	1.95	25.00	9.55	1.99	−0.63	.53
Months spent with trusted caregiver	18.38	10.75	2.35	17.50	8.64	1.93	−0.29	.78
Baseline/pretest								
Global quality	34.46	2.52	0.51	35.21	2.43	0.50	−1.05	.30
Sensitivity	4.60	0.83	0.17	4.98	0.66	0.13	−1.75	.09
Children’s wellbeing	4.50	0.77	0.15	4.50	0.35	0.07	0.01	.99
Posttest								
Global quality	35.92	3.05	0.62	34.75	3.44	0.70	1.24	.22
Observed sensitivity	4.53	0.81	0.17	4.75	0.86	0.18	−0.91	.37
Children’s wellbeing	4.80	0.42	0.08	4.73	0.44	0.09	−0.53	.60

### Procedure

All procedures were carried out with the adequate understanding and written informed consent of caregivers and parents. Ethical approval for this study was provided by the Leiden Institute of Education and Child Studies. There was no conflict of interest for any of the authors. The first author takes responsibility for the integrity and the accuracy of the data analysis.

During baseline, each setting was visited by an observer who spent a morning in the childcare homes to administer the Caregiver Interaction Scale (CIS; Arnett 1989) and the Infant Toddler Child Care Home Observation for Measurement of the Environment inventory (IT-CC-HOME; Caldwell and Bradley 2003). After the baseline visit, caregivers scoring low on sensitivity (CIS ≤ 3) were randomly assigned to either the control or the intervention group. All 48 caregivers received a pretest visit, in which the observer videotaped caregivers and children during episodes of regular childcare activities at predetermined time points (unstructured episodes) and structured play episodes of each 5 min.

Caregivers in the intervention group received six home visits and, parallel in timing, caregivers in the control group received six telephone calls. Post-test visits took place approximately 6 months after baseline (*M* = 5.92, *SD* = 1.14). Again, the IT-CC-HOME was administered and unstructured and structured play episodes were videotaped. All videotaped episodes were rated afterwards on children’s wellbeing and caregiver sensitivity by coders who were unaware of the experimental condition and who met the criteria to reliably assess these scales. To obtain independency in ratings, observers who visited the childcare setting did not rate children’s wellbeing or caregivers’ sensitivity in this specific setting, and coders who rated the pretest child fragments did not rate caregiver fragments or the fragments from the posttest, and vice versa. For the same reason, observers visited a specific childcare setting only once.

#### Intervention Program

*VIPP*-*CC.* The Video-feedback Intervention to promote Positive Parenting and Sensitive Discipline (VIPP-SD, Juffer et al. 2008) was adapted for implementation in home-based childcare: the Video-feedback Intervention to promote Positive Parenting: ChildCare (VIPP-CC). In the VIPP-CC program, the caregiver and the children are videotaped during daily situations in childcare (for example mealtime or structured playing) during short episodes of 10–30 min. To capture a more natural interaction, the intervener does not give any advice, tips or comments during the videotaping. Caregivers are encouraged to react to the children the way they normally do. In the period between the home visit and the first intervention session the intervener carefully reviews the videotape and prepares comments on the children’s and caregiver’s behavior on the video. During the next visit the intervener shows the whole videotape to the caregiver, while repeating and discussing the selected fragments. The intervention trajectory is—like the VIPP-SD—divided into three phases, which all consist of two sessions. During every session there are two intervention themes, one directed at sensitivity and one directed at discipline, based on the video fragments.

In the first phase, interveners build a relationship with the caregiver, with an emphasis in their video-feedback on child behavior. The themes of the first two sessions focusing on sensitivity (S) are (S1) exploration versus attachment behavior: showing the difference between the child’s contact seeking behavior and play and explaining the differential responses needed from the caregiver, (S2) ‘speaking for the child’: promoting the accurate perception of children’s signals by verbalizing their facial expressions and nonverbal cues. For discipline (D), the themes for these two sessions are (D1) inductive discipline and distraction: recommending induction and distraction as non-coercive responses to difficult child behavior and (D2) positive reinforcement: praising the child for positive behavior and ignoring negative attention seeking. The second phase focuses at improving caregiver behavior by showing at what moments strategies work. The sensitivity themes of the two sessions in this phase focus on (S3) the sensitivity chain: explaining the relevance of prompt and adequate responding to the child’s signals, and (S4) sharing emotions: showing and encouraging parents’ affective attunement to the emotions of their child. For discipline, these two sessions focus on (D3) the use of a sensitive time-out: a short break to calm down and (D4) empathy: showing understanding for the child. The third phase consists of two booster sessions in which all feedback and information is reviewed. At the end of the intervention program, caregivers receive a brochure with information on key issues discussed during the home visits (for a detailed description of the VIPP-SD, see Juffer et al. 2008). To be able to discuss all these themes, the tasks during the video-taping were selected for a specific theme. For example, the sensitivity chain was discussed during meal time, where caregivers and children normally react to each other, while positive reinforcement (theme discipline) was discussed during a clean up task.

To implement the original VIPP-SD to childcare, we adapted the program for caregivers taking care of a group of children by slightly modifying the procedure and materials of the home visits, as the situation in home-based childcare differs from the home situation (e.g. more than one child present, professional childcare). In the VIPP-SD, interveners first videotaped structured play sessions (for about half an hour) and then subsequently discussed the videotaped episodes from the last visit (for about an hour) based on prepared comments (script). In the VIPP-CC, interveners first videotaped the structured play sessions and then left the home, allowing caregivers and children to have a quiet lunch. After the caregivers put (some of) the children into bed, interveners returned and discussed the videotaped episodes from the last visit. Furthermore, the ‘speaking for the child’ was not only directed to one child at a time, but also to the entire group of children (‘speaking for the children’), emphasizing caregivers’ attention for the signals of all children present. In addition, the toys that were used during structured play situations were adapted for a group setting, for example by using a big box of Duplo bricks and large story books.

Interveners (*n* = 7) had comparable backgrounds in terms of education. They were all graduate students at the Centre of Child and Family Studies, who were first trained on the VIPP-SD during a full-time week workshop by one of the VIPP-SD experts from the Centre of Child and Family Studies, including home assignments which were provided with feedback from the VIPP-SD expert. After this training, interveners received further training on the adapted VIPP-CC. During the intervention period, four feedback sessions were held, in which video-taped structured play situations and scripts were discussed, as well as how to build and obtain a professional relationship with the caregiver. We found no intervener effects.

#### Control Group

In order to keep in contact with all caregivers and to prevent attrition, caregivers in the control group received a dummy intervention (Juffer et al. 2005). Parallel to the intervention sessions, caregivers in the control group received six telephone calls. During these semi-structured interviews (scripts), caregivers were invited to talk about general developmental topics (e.g. eating, talking, playing). These six telephone calls, which lasted between 15 min and half an hour, were conducted by the same interveners who visited the intervention group. Interveners were trained prior to the study. The control group received no advice or information about sensitivity or child development. If caregivers would ask for advice or information, interveners were instructed to offer referrals to other services. None of the interveners was asked for advice. To attain treatment protocol adherence, four feedback sessions were held during the intervention period, in which the progress of the phone calls was discussed.

### Materials

#### Selection

For selection purposes, caregiver sensitivity in the group setting was examined by direct observation using the CIS. The CIS consists of 26 items; for each item a score is given from 1 (not applicable) to 4 (very applicable). In a Dutch study (Van IJzendoorn et al. 1998), two dimensions were found: sensitivity (14 items) and authoritarian caregiving (12 items). In the study reported here, the subscale ‘sensitivity’ was used, because of its close link with the aim of the intervention. Example items are ‘Speaks warmly to the children’ and ‘Listens attentively when children speak to her’. Prior to the data collection, ten observers were trained and became reliable on the CIS, Internal consistency (Cronbach’s alpha) of this scale was .84. Mean intra-class correlations of the observers (two-way mixed, absolute agreement) was .80 (range .78–.84).

#### Children’s Wellbeing

During pretest and posttest, three unstructured episodes of each 8 min (e.g., lunch, free play) were videotaped to code children’s wellbeing. Coding of videotaped episodes took place by means of the Laevers Wellbeing Scale (2003) that was adapted and validated by the Dutch Consortium for Child Care Research for infants and toddlers in childcare (NCKO; De Kruif et al. 2007). In this scale, wellbeing is defined as a general positive state of a child (Balledux 2002) and the extent to which children feel safe, self-confident, relaxed and are enjoying the activities in which are they are involved. The wellbeing scale contains several indicators of the child’s wellbeing, such as pleasure, self-confidence, and relaxation. Wellbeing scores are presented on a seven-point scale, ranging from (1) a very low wellbeing (signals of discomfort are clearly present, e.g., crying, screaming) to (7) a very high wellbeing (signals of comfort are clearly present, e.g., enjoyment, smiling). Scores were aggregated across the time periods.

Six observers were trained to reliably assess the children’s wellbeing. All observers met the criterion of reliability on the same dataset: mean intra-class correlation (two-way mixed, absolute agreement) was .73 (range from .70 to .78). Internal consistency of the intervals was .86.

#### Global Quality of Childcare

The IT-CC-HOME (Caldwell and Bradley 2003) is designed to measure the quality and quantity of stimulation and support available to a child in the childcare home environment, and covers various domains of childcare: responsivity, acceptance, organization, learning materials, involvement, and variation. A positive (1) or a negative (0) score is achieved for each of the 43 items. Two items were deleted from the scale: item 21 ‘Child gets out of house at least four times a week’ and item 42 ‘Caregiver and child visit or receive visits from neighbor or friends once a month or so’. These items were not applicable to the Dutch situation, because in the Netherlands children attend home-based childcare on average 2 or 3 days a week, in contrast to other countries. The total IT-CC-HOME score is a summation across the 41 item scores (0 or 1). Internal consistency (Cronbach’s alpha) of this scale was .60. Ten observers were trained prior to the study. After a general introduction, observers visited at least four caregivers in pairs to complete the IT-CC-HOME. Each observation was followed by an item-by-item debriefing with the trainer. Prior to this debriefing, interrater reliability was established to a criterion of 80 % agreement.

#### Caregiver Sensitivity

During pretest and posttest, three unstructured episodes of each 10 min (e.g., lunch, free play) and two structured play episodes of each 5 min were videotaped to code caregiver sensitivity. Both structured situations consisted of 10 min play with Duplo bricks or a car rollercoaster. Caregivers were asked to play with the children as they would normally do. Coding of videotaped episodes took place by means of a scale developed and validated by the Dutch Consortium for Child Care Research (NCKO; Helmerhorst et al. 2014). This group rating scale is based on scales developed to measure sensitivity in a parent–child context (Ainsworth et al. 1974; Erickson et al. 1985). Sensitivity ratings are presented on a seven-point scale, ranging from (1) very low sensitivity to (7) very high sensitivity. Five observers were trained and became reliable on the same dataset to assess caregivers’ sensitivity. Mean intra-class correlations (two-way mixed, absolute agreement) was .73 (range .69–.75). Internal consistency of this scale was .74 (pretest) and .83 (posttest). During data collection, sensitivity of ten caregivers was doubly coded, resulting in an intra-class correlation of .95. Because scores on the three unstructured episodes and the two structured play episodes did not differ significantly, mean sensitivity scores were aggregated across these five episodes.

### Data Analysis

To test whether changes occurred in children’s wellbeing scores, repeated measures ANOVA’s were conducted controlling for the pretest measures. In addition, quality measures (global quality and sensitivity) were included as covariates into the repeated measures ANOVA to test whether childcare quality influenced the change in wellbeing over time. Missing data were present at item level only: these missing item scores were filled in with the person mean on the other items from that particular scale. To test the effect of months spent with the caregiver, we used a residual variable representing the number of months children attended this particular childcare setting controlled for the age of the children. Seven children had missing data on ‘months spent with the caregiver’. These missing values were imputed using multiple imputation with predictive mean matching in SPSS. Analysis results were pooled using the standard procedure in SPSS, which is based on the rules described by Rubin (1987).

## Results

### Descriptives

Descriptive statistics and correlations of the baseline, pretest and posttest measures are shown in Tables 1 and 2. Children’s wellbeing ranged from 2.50 to 5.58 during the pretest (intervention group *M* = 4.5. *SD* = 0.77; control group *M* = 4.50, *SD* = 0.42), during the posttest wellbeing ranged from 3.38 to 5.5 in the intervention group (*M* = 4.8, *SD* = 0.42) and from 4.08 to 5.67 in the control group (*M* = 4.73, *SD* = 0.44). No significant differences were present between the intervention group and the control group during both pre- and posttest (without taking any covariates into account).

**Table 2:** Pearson correlations between baseline/pretest and posttest

	Baseline/pretest			Posttest		
	Global quality	Sensitivity	Children’s wellbeing	Global quality	Sensitivity	Children’s wellbeing
Baseline/pretest						
Global quality	–	0.33	0.02	0.56**	0.34	−0.18
Sensitivity	0.04	–	0.08	0.32	0.43*	0.27
Children’s wellbeing	−0.16	−0.17	–			0.10
Posttest						
Global quality	0.36	0.50*	0.07	–	0.40	−0.35
Sensitivity	0.55**	0.40	0.02	0.36	–	0.07
Children’s wellbeing	0.35	−0.08	0.35	0.33	0.41*	–

Correlations within the intervention group are displayed below the diagonals and correlations within the control group are displayed above the diagonals

* *p* < .05, ** *p* < .01

For the intervention group, children’s wellbeing in the posttest was positively associated with caregiver sensitivity in the posttest (*r* = .41, *p* < .05). In addition, global quality during baseline was positively associated with caregiver sensitivity during the posttest (*r* = .55, *p* < .01), and global quality during the posttest was positively associated with caregiver sensitivity during the pretest (*r* = .50, *p* < .01). In the control group, global quality was positively associated during baseline and posttest (*r* = .56, *p* < .01). Also, observed sensitivity during pretest and posttest were positively associated (*r* = .43, *p* < .05).

For months spent with the caregiver, again no difference was present between the intervention group and the control group. Prior to the intervention, there were two significant associations between months spent with the caregiver and quality of care: caregivers were more sensitive to children who were in their care for a shorter period of time (*r* = −.31, *p* < .05), and global childcare quality was higher when children spent a longer period of time with that specific caregiver (*r* = .30, *p* < .01).

### Intervention Effect

To test whether the wellbeing of the children improved in the intervention group as compared to the control group, repeated measures ANOVA’s were conducted. A main effect was present for time [*PillaisF*(1, 45) = 7.75, *p* < .01, *η*_*p*_^2^ = .15], indicating an overall increase in children’s wellbeing from the pretest to the posttest in both the intervention group and the control group. No significant main effect was present for group [*PillaisF*(1, 45) = 0.07, *p* = .79, *η*_*p*_^2^ < .01]. In addition, no significant interaction effect was found [*PillaisF*(1, 45) = 0.13, *p* = .72, *η*_*p*_^2^ < .01, *d* = 0.11].

To test whether caregiver sensitivity and/or global quality moderated the change in wellbeing over time, the quality measures were added to the repeated measures ANOVA and the interactions between quality and time were investigated. We found no significant interaction effects [pretest global quality: *PillaisF*(1, 44) = 1.11, *p* = .30, *η*_*p*_^2^ = .03; posttest global quality: *PillaisF*(1, 44) = 0.14, *p* = .70, *η*_*partial*_^2^ < .01; pretest sensitivity: *PillaisF*(1, 44) = 1.03, *p* = .32, *η*_*p*_^2^ = .02; posttest sensitivity: *PillaisF*(1, 44) = 0.24, *p* = .63, *η*_*p*_^2^ < .01].

### Months Spent with Trusted Caregiver

On average, children were 25.89 months old during the pretest (*SD* = 9.49), ranging from 6 months of age to 42 months of age. The time they had spent with the caregiver ranged from 3 to 36 months (*M* = 17.95, *SD* = 9.67). No differences in months spent with the caregiver were present between the intervention group and the control group.

To test whether time spent with a trusted caregiver (months spent with the caregiver corrected for the age of the children) acted as a moderator of the intervention effect, a hierarchical regression analyses was conducted (Table 3). In the first step we included children’s wellbeing during the pretest, which did not predict children’s wellbeing during the posttest (*B* = 0.18, *SEB* = 0.10, *t* = 1.77, *p* = .08). In the second step, there were no main effects of (intervention or control) group (*B* = 0.07, *SEB* = 0.13, *t* = 0.52, *p* = .60) or time spent with the caregiver (*B* = 0.02, *SEB* = 0.07, *t* = 0.37, *p* = .79). In the third step, a significant interaction effect was present between group and time spent with the caregiver (*B* = 0.27, *SEB* = 0.13, *t* = 2.14, *p* < .05, *R*^2^ = .18).

**Table 3:** Hierarchical Regressions in Home-based Child Care: pooled data: predicting posttest wellbeing

	*B*	*SEB*	*t*	Fraction missing info	Relative increase variance	Relative efficiency	*R* ^2^
Step 1							0.06
Constant	3.96	0.46	8.54	0.00	0.00	1.00	
Pretest: Children’s wellbeing	0.18	0.10	1.77	0.00	0.00	1.00	
Step 2							0.07
Group	0.07	0.13	0.52	0.04	0.04	0.99	
Months with trusted caregiver	0.02	0.07	0.37	0.14	0.15	0.97	
Step 3							0.18
Group*Months with trusted caregiver	0.27	0.13	2.14*	0.11	0.12	0.98	

* *p* < .05

By adding the childcare quality measures to this regression analysis (change scores from pretest to posttest controlled for pretest scores), no significant interaction effects emerged with time spent with the caregiver for caregiver sensitivity (*B* = 0.09, *SEB* = 0.37, *t* = 0.23, *p* = .82) or global childcare quality (*B* = −0.05, *SEB* = 0.64, *t* = −0.08, *p* = .94).

**Fig. 2:**
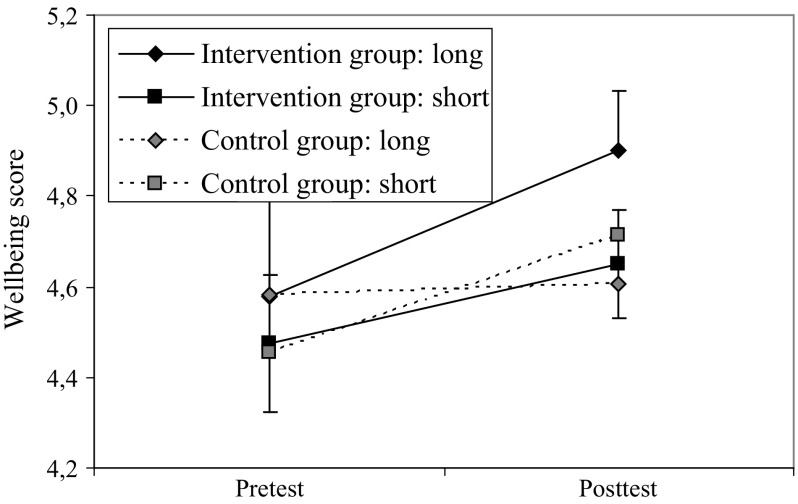
Children’s wellbeing scores in the Intervention Group and the Control group during Pretest and Posttest, split for Months with trusted caregiver (long = above median, short = below median)

To illustrate the interaction between the intervention and time spent with the trusted caregiver, we dichotomized time in childcare by using a median-split procedure (median standardized residual at 0.26, all of these children spent more than 80 % of their lives in the care of this caregiver, for example 30 months of their lives at the age of 33 months). Four groups are presented in Fig. 2: children who were less familiar with the caregiver and children who were more familiar with the caregiver—split for the intervention group and the control group. As is shown in Fig. 2, the wellbeing scores of children who were less familiar with the caregiver increased from pretest to posttest in both the intervention and the control group (cubes). A different pattern of wellbeing scores was present in the group of children who were more familiar with the caregiver (diamonds). While in the control group these children showed stable scores in wellbeing from pretest to posttest, children in the intervention group showed an increase in wellbeing over time. In the intervention group, children’s posttest wellbeing scores were higher when children were more familiar with the caregiver than when children were less familiar with the caregiver [*pooled t* (250) = −1.94, *p* = .05]. In the control condition, these groups were similar [*pooled t* (122) = 0.91, *p* = .37].

## Discussion

Previous studies in families have shown the effectiveness of the VIPP-SD in changing children’s behavior, especially in decreasing children’s externalizing behavior problems in high risk samples (Klein Velderman et al. 2006a, b; Bakermans-Kranenburg et al. 2008; Van Zeijl et al. 2006). No studies yet focused on the effectiveness of the VIPP-SD in improving children’s wellbeing. In the current paper, we tested the effect of this intervention on children’s wellbeing in home-based childcare. In a previous paper an intervention effect was shown of the VIPP-CC on global childcare quality (*η*_*p*_^2^ = 0.76; Groeneveld et al. 2012). Although we did not find an overall intervention effect on child wellbeing, a significant interaction effect with months spent with the caregiver was present. Children who attended childcare for a shorter period of time showed an increase in wellbeing scores (intervention group and control group), but for the group of children who attended childcare for a longer period of time, wellbeing increased only in the intervention group (and not in the control group).

### Increase in Children’s Wellbeing

In the current study, we found an increase in children’s wellbeing during the study period, both for children in the intervention group and the control group. No other studies in childcare address children’s wellbeing over a longer period of time, so it is unknown from the literature whether children’s wellbeing normally increases or decreases over time. In the NICHD study (2001), the frequency of observed positive social play of children remained the same from 24 to 36 months of age, but negative peer play decreased over time. In a study focusing on stress levels of children, it was shown that cortisol levels of children in childcare decrease over time: children who just attended childcare centers (aged 11–20 months) showed higher cortisol levels than 5 months later (Ahnert et al. 2004), indicating that children might be more at ease at childcare over time.

The fact that we did not find a stronger increase in wellbeing in the total intervention group compared to the control group may be explained by the timing of our posttest. Effects of interventions may lie dormant directly after the intervention (sleeper effect), but may become noticeable later on. In the current study, the posttest took place 2 weeks after the last intervention session. Although global childcare quality did improve in the intervention group (*d* = 0.76); Groeneveld et al. 2012) children might not yet have become accustomed to this improvement. In the Netherlands, most children visit childcare for only 2 or 3 days a week. An effect on children’s wellbeing scores might have been detected if the posttest had taken place later on.

Previous studies on the effect of the VIPP-SD were mainly conducted in families with difficulties (e.g., with insecure attachment relationships, insensitive parents, maternal mental health problems, or child behavior problems). The current study attempts to stay close to such “at risk” situations by focusing on caregivers scoring relatively low on sensitivity (CIS score < 3.0). However, there might have been a ceiling effect, since the average wellbeing and sensitivity score of the included children and caregivers was still quite high, compared to previous Dutch childcare studies (Groeneveld et al. 2010; Helmerhorst et al. 2014). The effect of the VIPP-CC might have been larger in a group of caregivers scoring lower on sensitivity during the pretest, or on children scoring low on wellbeing during this pretest.

### Months Spent with Trusted Caregiver

We explored whether months spent with a trusted caregiver may be an important moderator of the intervention effect on children’s wellbeing. Although the relation between the number of hours per week children spend in childcare and their social-emotional development has been studied (Sylva et al. 2007; NICHD Early Child Care Research Network 1998, 2001), the impact of months spent with the caregiver as a proportion of children’s age is yet unexplored. It is plausible that the effect of an intervention is different for children who are in the care of a particular caregiver for a large part of their lives compared to children who are less familiar with a particular caregiver. This might be especially important in home-based childcare, were children are taken care of by the same caregiver every day.

Prior to the intervention, we found no association between months spent with the trusted caregiver and the wellbeing of the children. We did find associations between quality of care and months spent with the caregiver: caregivers were less sensitive to children who were in their care for a longer period of time, but offered higher global childcare to this group of children (compared to children who had spent less time with this caregiver). This difference in global childcare quality might be due to caregivers being more able to adjust the childcare environment (e.g., play materials, outings) to children they are more familiar with. In addition, an interaction effect with (the intervention or control) group was present. Wellbeing scores of children who visited the caregiver for a shorter period of time increased from pretest to posttest in both the intervention and the control group, but a different pattern of wellbeing scores was present in the group of children who visited the caregiver for a longer period of time. While in the control group these children showed stable scores in wellbeing from pretest to posttest, children in the intervention group showed an increase in wellbeing over time. Thus, the VIPP-CC seems most effective in children who have been cared for by a caregiver for a longer period of time. A possible explanation is that the familiarity of the childcare setting is the first step in order to profit from the intervention. The childcare context is different from the children’s home situation, with another adult taking care of them in a new environment, with an unfamiliar group of children, and without the presence of their parents. A change in childcare quality might not have a large impact on the wellbeing of these children, since they are still trying to get used to the childcare environment with an unfamiliar caregiver and unfamiliar children. When children are accustomed to the childcare environment, subtle changes in childcare quality of this environment might have more effect on their wellbeing compared to their peers who are not accustomed to the childcare environment. Thus, our results indicate that the VIPP-CC seems most effective when it is implemented in childcare settings where children are accustomed to the childcare environment. It should be noted however that this interaction effect needs to be confirmed in larger samples, and preferably in childcare centers as well.

### Limitations

The sample size of this study is relatively small, which may have resulted in a lack of statistical power to detect moderation effects. As a result, we were limited in the number of moderators we could include in the study. We selected one target child per caregiver, which reduced our sample size, but had the advantage that all caregivers and children in our sample were from different childcare settings. Since the children were randomly selected prior to the intervention, we do not expect that we only selected children who would have shown more or less improvement than the other children in that setting. Besides sample size, another limitation of the study is the relatively high level of childcare quality and wellbeing scores prior to the intervention. Although caregivers with very high scores on caregiver sensitivity were excluded, scores on childcare quality and children’s wellbeing were still relatively high. A ceiling effect is possible and might have decreased the intervention effects.

Furthermore, not all caregivers responded positively to our invitation to participate in our research, which might have resulted in a selection bias. In addition, 17 caregivers (26 %) dropped out after the selection phase. However, attrition seems unavoidable in intervention studies in childcare, even during the intervention phase. For example in the Family-to-Family study, 27 % of the caregivers dropped out during the intervention phase (Kontos et al. 1996). In the individualized REACH program, in total 43 % of the caregivers dropped out (Espinosa et al.^1999^). In our study, only two caregivers dropped out during the intervention phase; one caregiver in the control group was unwilling to participate in the posttest and one caregiver in the intervention group did finish the intervention, but with a different group of children than during the pretest, due to the target child moving to a different setting.

In addition, the non-inclusion of a follow-up is a limitation of this study. We do not know whether children’s wellbeing in the intervention will improve in the long run, nor do we know whether the reported interaction effect will remain over time.

## Conclusion

The current study revealed that children’s wellbeing scores in home-based childcare increase over time. For children who have been cared for by the same trusted caregiver for a longer period of time, the VIPP-CC was effective in enhancing their wellbeing.

## References

[CR1] Aguirre BE, Marshall MG (1988). Training family day care providers using self-study written video materials. Child & Youth Care Quarterly.

[CR2] Ahnert L, Gunnar MR, Lamb ME, Barthel M (2004). Transition to child care: Associations with infant–mother attachment, infant negative emotion, and cortisol elevations. Child Development.

[CR3] Ainsworth MDS, Bell SM, Stayton D, Richards MP (1974). Infant–mother attachment and social development. The introduction of the child into a social world.

[CR4] Ainsworth MDS, Blehar MC, Waters E, Wall S (1978). Patterns of attachment: A psychological study of the strange situation.

[CR400] Amato PR, Keith B (1991). Parental divorce and the well-being of children: A meta-analysis. Psychological Bulletin.

[CR5] Arnett J (1989). Caregivers in day-care centers: Does training matter?. Journal of Applied Developmental Psychology.

[CR6] Bakermans-Kranenburg MJ, Van IJzendoorn MH, Juffer F (2003). Less is more: A meta-analyses of sensitivity and attachment interventions in early childhood. Psychological Bulletin.

[CR40] Van Bakermans-Kranenburg MJ, Van IJzendoorn MH, Pijlman FTA, Mesman J, Juffer F (2008). Experimental evidence for differential susceptibility: Dopamine D4 receptor polymorphism (DRD4 VNTR) moderates intervention effects on toddlers’ externalizing behavior in a randomized controlled trial. Developmental Psychology.

[CR7] Balledux, M. (2002). Werken aan welbevinden: Het evaluatie-instrument voor de kinderopvang opnieuw bekeken. *[Working on wellbeing: a closer look at the evaluation instrument for childcare settings]* Utrecht: NIZW.

[CR300] Barandiaran A, Muela A, de Arana EL, Larrea I, Vitoria JR (2015). Exploratory behaviour, emotional wellbeing and childcare quality in preschool education. Anales de Psicología.

[CR8] Barnett WS, Junga K, Yarosza DJ, Thomas J, Hornbeck A, Stechuk R, Burns S (2008). Educational effects of the tools of the mind curriculum: A randomized trial. Early Childhood Research Quarterly.

[CR9] Bowlby J (1969). Attachment and loss: Vol. 1. Attachment.

[CR500] Bradley RH, Vandell DL (2007). Child care and the well-being of children. Archives of Pediatrics & Adolescent Medicine.

[CR10] Caldwell BM, Bradley RH (2003). Home observation for measurement of the environment: Administration manual.

[CR700] Clarke-Stewart KA, Hayward C (1996). Advantages of father custody and contact for the psychological well-being of school-age children. Journal of Applied Developmental Psychology.

[CR200] Davis E, Priest N, Davies B, Sims M, Harrison L, Herrman H (2010). Promoting children’s social and emotional wellbeing in childcare centers within low socioeconomic areas: Strategies, facilitators and challenges. Australasian Journal of Early Childhood.

[CR11] De Kruif REL, Vermeer HJ, Fukkink RG, Riksen-Walraven JMA, Tavecchio LWC, Van IJzendoorn MH (2007). De nationale studie pedagogische kwaliteit kinderopvang: Eindrapport project 0 en 1 [The national study childcare quality: Final report project 0 and 1].

[CR12] De Schipper JC, Van IJzendoorn MH, Tavecchio LWC (2004). Stability in center day care: Relations with children’s well-being and problem behavior in day care. Social Development.

[CR13] Downer JT, Pianta RC, Fan X, Hamre BK, Mashburn A, Justice L (2011). Effects of web-mediated teacher professional development on the language and literacy skills of children enrolled in prekindergarten programs. NHSA Dialog: A Research-to-Practice Journal for the Early Childhood Field.

[CR14] Erickson, M. F., Sroufe, L. A., & Egeland, B. (1985). The relationship between quality of attachment and behavior problems in preschool in a high risk sample. *Monographs of the society for research in child development,**50,* 147–166. 4069126

[CR15] Espinosa L, Mathews MC, Thornburg KR, Ispa J (1999). Training and rural child care providers: Results of Project Reach. NHSA Dialog.

[CR16] Girard L, Girolametto L, Weitzman E, Greenberg J (2011). Training early childhood educators to promote peer interactions: Effects on children’s aggressive and prosocial behaviors. Early Education and Development.

[CR17] Girolametto L, Weitzman E, Greenberg J (2004). The effects of verbal support strategies on small-group peer interactions. Language, Speech, and Hearing Services in Schools.

[CR18] Groeneveld MG, Vermeer HJ, Van IJzendoorn MH, Linting M (2010). Stress, cortisol, and well-being of caregivers and children in home-based child care: A case for differential susceptibility. Child: Care Health & Development.

[CR19] Groeneveld MG, Vermeer HJ, Van IJzendoorn MH, Linting M (2012). Enhancing home-based child care quality through video-feedback intervention: A randomized controlled trial. Journal of Family Psychology.

[CR20] Helmerhorst KOW, Riksen-Walraven MJ, Vermeer HJ, Fukkink RG, Tavecchio LWC (2014). Measuring the interactive skills of caregivers in child care centers: Development and validation of the caregiver interaction profile scales. Early Education and Development.

[CR21] Howard J, McInnes K (2012). The impact of children’s perception of an activity as play rather than not play on emotional well-being. Child: Care Health, and Development.

[CR22] Juffer, F., Bakermans-Kranenburg, M. J., & Van IJzendoorn. M. H. (2009). Attachment-based intervention: Heading for evidenced-based ways to support families. In *ACAMH Occasional Papers NO.29, Attachment: Current Focus and Future Directions* (pp. 47–57).

[CR23] Juffer F, Bakermans-Kranenburg MJ, Van IJzendoorn MH (2005). The importance of parenting in the development of disorganized attachment: Evidence from a preventive intervention study in adoptive families. Journal of Child Psychology and Psychiatry.

[CR24] Juffer F, Bakermans-Kranenburg MJ, Van IJzendoorn MH (2008). Promoting positive parenting: An attachment-based intervention.

[CR25] Kalinauskiene L, Cekuoliene D, Van IJzendoorn MH, Bakermans-Kranenburg MJ, Juffer F, Kusakovskaja I (2009). Supporting insensitive mothers: The Vilnius randomized control trial of video feedback intervention to promote maternal sensitivity and infant attachment. Child: Care Health & Development.

[CR26] Klein Velderman M, Bakermans-Kranenburg MJ, Juffer F, Van IJzendoorn MH (2006). Effects of attachment-based interventions on maternal sensitivity and infant attachment: Differential susceptibility of highly reactive infants. Journal of Family Psychology.

[CR27] Klein Velderman M, Bakermans-Kranenburg MJ, Juffer F, Van IJzendoorn MH, Mangelsdorf SC, Zevalkink J (2006). Preventing preschool externalizing behavior problems through video-feedback intervention in infancy. Infant Mental Health Journal.

[CR28] Kontos S, Howes C, Galinsky E (1996). Does training make a difference to quality in family child care?. Early Childhood Research Quarterly.

[CR29] Laevers F (2000). Forward to basics: Deep-level-learning and the experiential approach. Early Years: An International Research Journal.

[CR30] NICHD Early Child Care Research Network (1998). Early child care and self-control, compliance, and problem behavior at 24 and 36 months. Child Development.

[CR31] NICHD Early Child Care Research Network (2001). Early child care and children’s peer interaction at 24 and 36 months. Child Development.

[CR32] NICHD Early Child Care Research Network (2002). Child-care structure → process → outcome: Direct and indirect effects of child-care quality on young children’s development. Psychological Science.

[CR33] Patterson GR (1982). Coercive family process.

[CR34] Riksen-Walraven M, Van IJzendoorn R, Tavecchio L, Riksen-Walraven M (2004). Pedagogische kwaliteit in de kinderopvang: Doelstellingen en kwaliteitscriteria. [Childcare quality: Objectives and criteria]. De kwaliteit van de Nederlandse kinderopvang [Quality of Dutch childcare].

[CR35] Rubin DB (1987). Multiple imputation for nonresponse in surveys.

[CR36] Rusby JC, Smolkowski K, Marquez B, Taylor TK (2008). A small-scale randomized efficacy trial of Carescapes: Enhancing children’s social development in child care homes. Early Childhood Research Quarterly.

[CR37] Snyder J, Low S, Schultz T, Barner S, Moreno D, Garst M, Leiker R, Swink N, Schrepferman L (2011). The impact of brief teacher training on classroom management and child behavior in at-risk preschool settings: Mediators and treatment utility. Journal of Applied Developmental Psychology.

[CR38] Statistics Netherlands, Cijfers kinderopvang eerste kwartaal van 2014, *Retrieved from* http://www.kinderopvangtotaal.nl/PageFiles/5572/001_1402563764428.pdf on July 9th 2015.

[CR39] Stein A, Woolley H, Senior R, Hertzmann L, Lovel M, Lee J (2006). Treating disturbances in the relationship between mothers with bulimic eating disorders and their infants: A randomized, controlled trial of video feedback. The American Journal of Psychiatry.

[CR550] Sylva K, Stein A, Leach P, Barnes J, Malmberg LE (2007). Family and child factors related to the
use of non-maternal infant care: An English study. Early Childhood Research Quarterly.

[CR600] Van IJzendoorn MH, Tavecchio LW, Stams GJJ, Verhoeven MJ, Reiling EJ (1998). Quality of center day care and attunement between parents and caregivers: Center day care in cross-national perspective. The Journal of Genetic Psychology.

[CR41] Van Zeijl J, Mesman J, Van IJzendoorn MH, Bakermans-Kranenburg MJ, Juffer F, Stolk MN (2006). Attachment-based intervention for enhancing sensitive discipline in mothers of 1- to 3-year-old children at risk for externalizing behavior problems: A Randomized controlled trial. Journal of Consulting and Clinical Psychology.

[CR42] Wasik BA, Bond MA, Hindman A (2006). The effects of a language and literacy intervention on head start children and teachers. Journal of Educational Psychology.

[CR43] Werner, C. D., Linting, M., Vermeer, H. J., & Van IJzendoorn, M. H. (2015). Do intervention programs in childcare promote the quality of caregiver–child interactions? A meta-analysis of randomized controlled trials. *Prevention Science*, 1–15. doi:10.1007/s11121-015-0602-7. 10.1007/s11121-015-0602-7 PMC4718933 26411312

